# Supplementary material for: A whole-genome CRISPR screen identifies the spindle accessory checkpoint as a locus of nab-paclitaxel resistance in a pancreatic cancer cell line
Source: Sci Rep. 2024 Jul 10;14:15912. doi: 10.1038/s41598-024-66244-1 (PMC11236977; doi:10.1038/s41598-024-66244-1)
Supplement: Supplementary file 1 — Supplementary Figures. [file 41598_2024_66244_MOESM1_ESM.pdf]

A

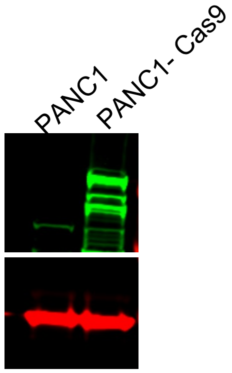

B

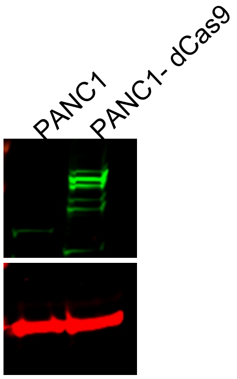

S1A, B

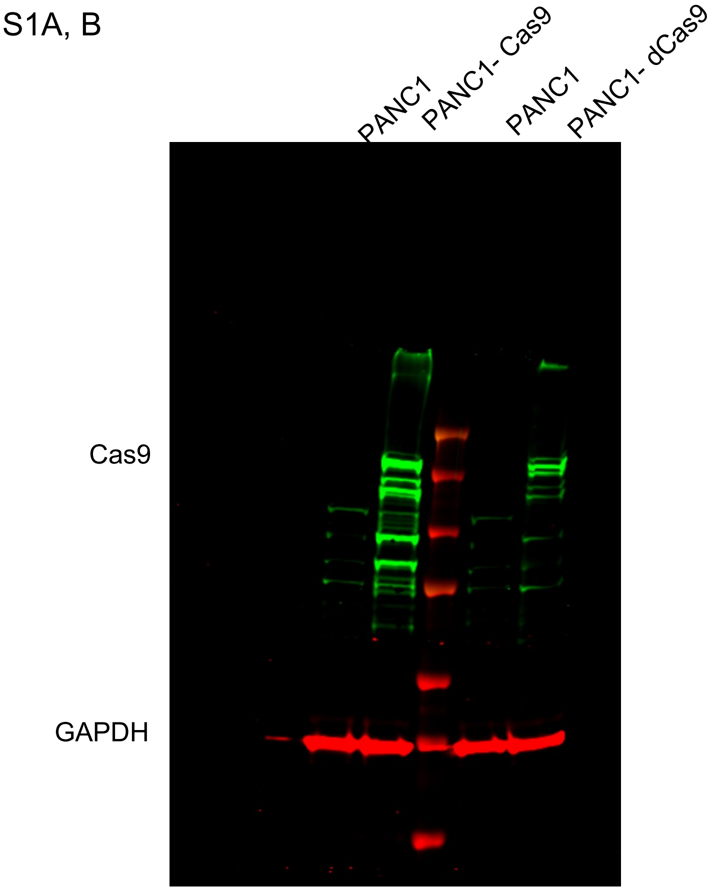

C

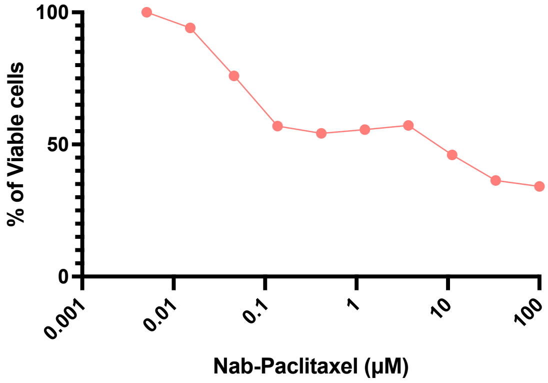

Figure S1. The stable expression of Cas 9 (A) and dCas9-KREB (B) is confirmed by Western blot. GAPDH is used as a control. Unprocessed western blot images for panel A and B shown below. (C) The proliferation of PANC-1 cells is significantly suppressed by 10 μM nab-paclitaxel treatment, ensuring an effective selection pressure for the genetic screen.

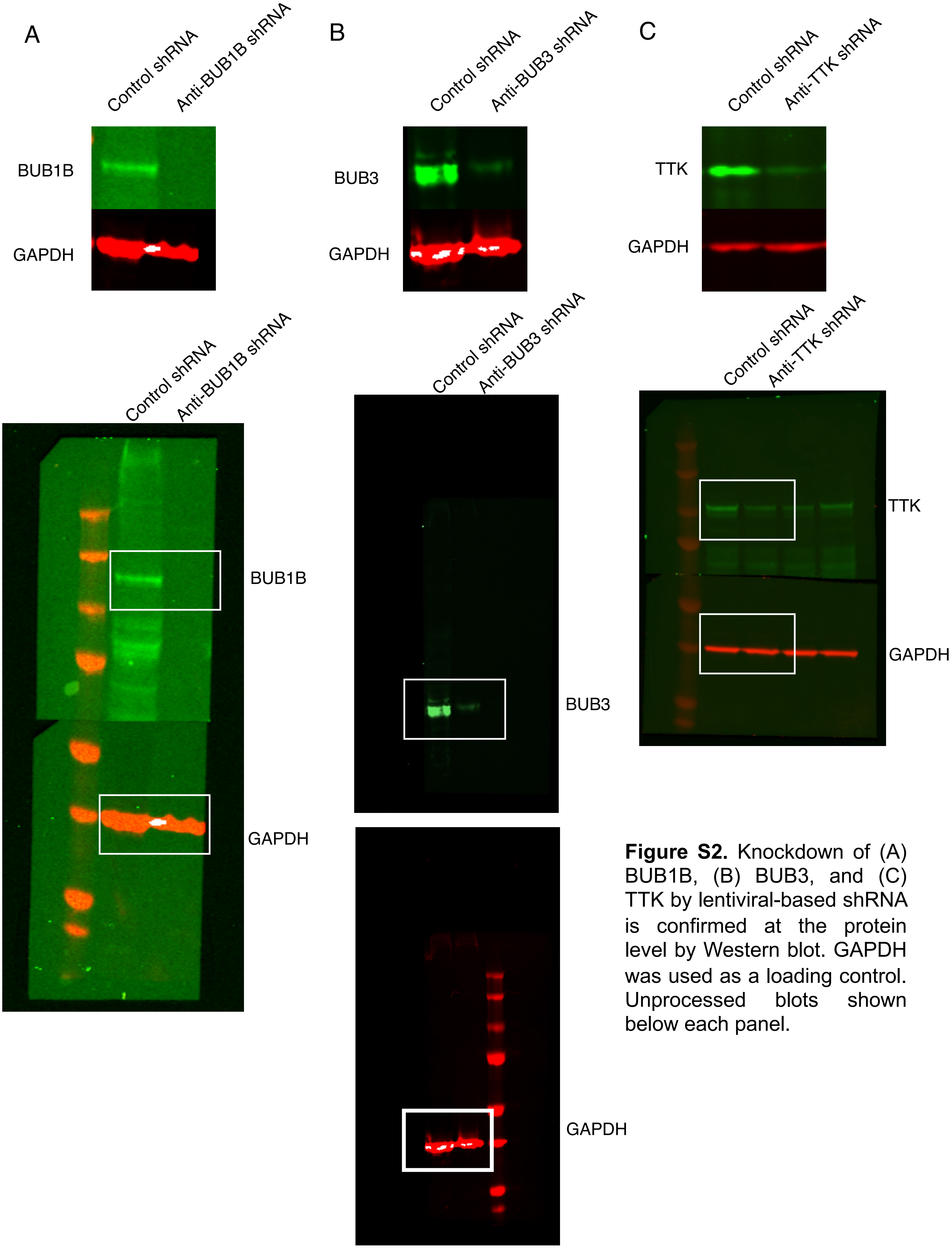

Figure S2. Knockdown of (A) BUB1B, (B) BUB3, and (C) TTK by lentiviral-based shRNA is confirmed at the protein level by Western blot. GAPDH was used as a loading control. Unprocessed blots shown below each panel.

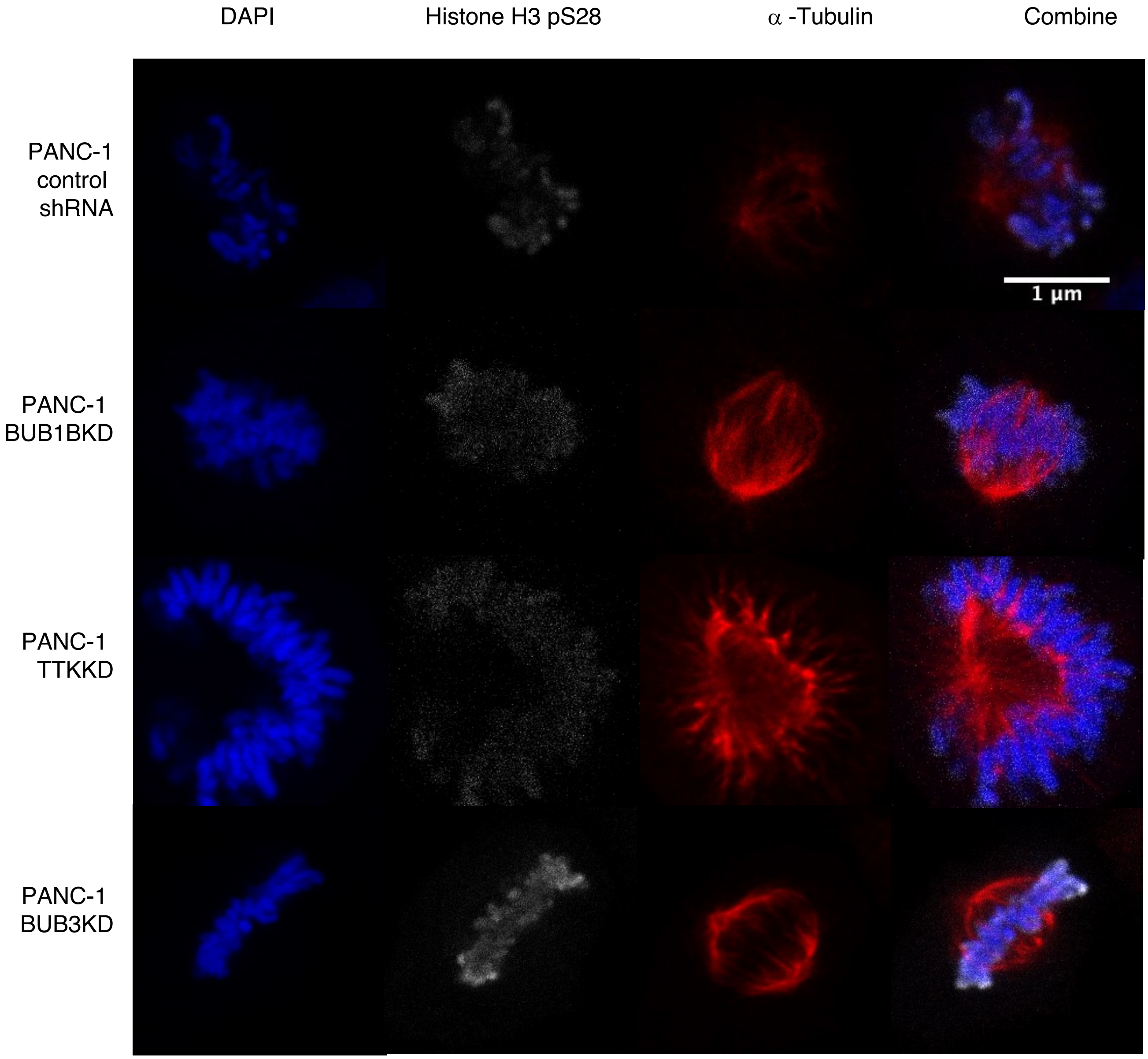

Figure S3. shRNA-transfected PANC-1 cells were immunostained for DAP1 (blue), Histone H3p28 (white), and α-tubulin (red). Scale bars: 1 μM.

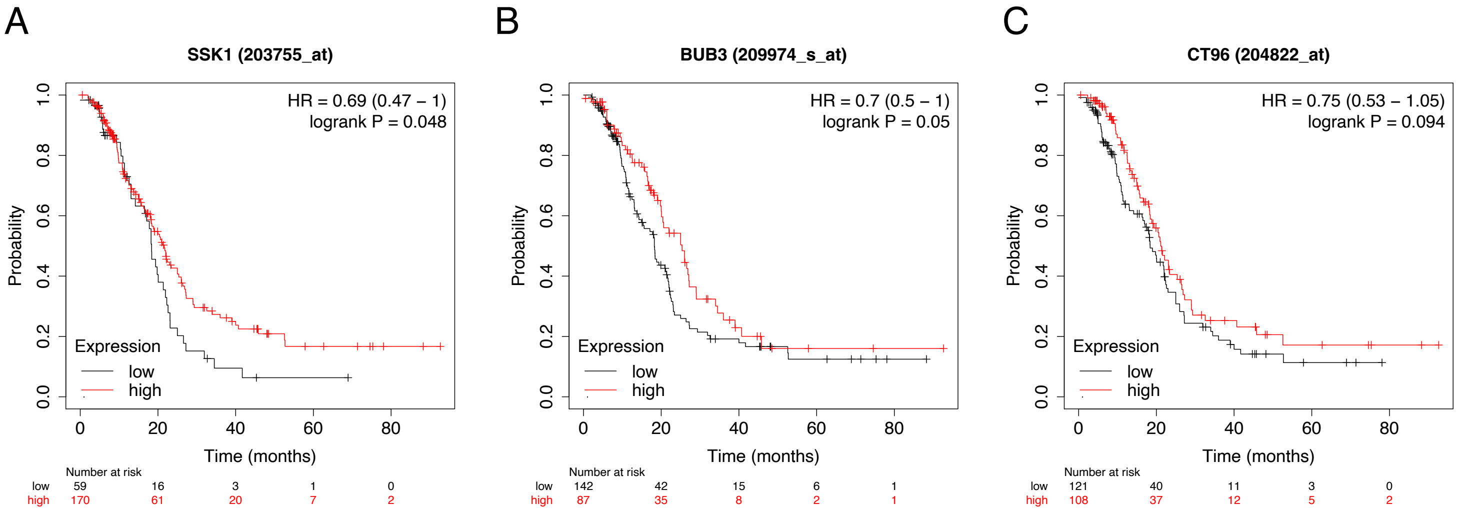

Figure S4. Relationship between the expression of BUB1B (SSK1), BUB3, and TTK (CT96) with overall survival in ovarian cancer patients using data from the KM plotter database (kmplot.com). Survival curves were plotted based on the mRNA values from about 250 ovarian cancer patients treated with paclitaxel (GSE14764). The findings showed that high expression levels of BUB1B, BUB3, or TTK were associated with better survival rates in ovarian cancer patients treated with paclitaxel compared to patients with low expression levels.

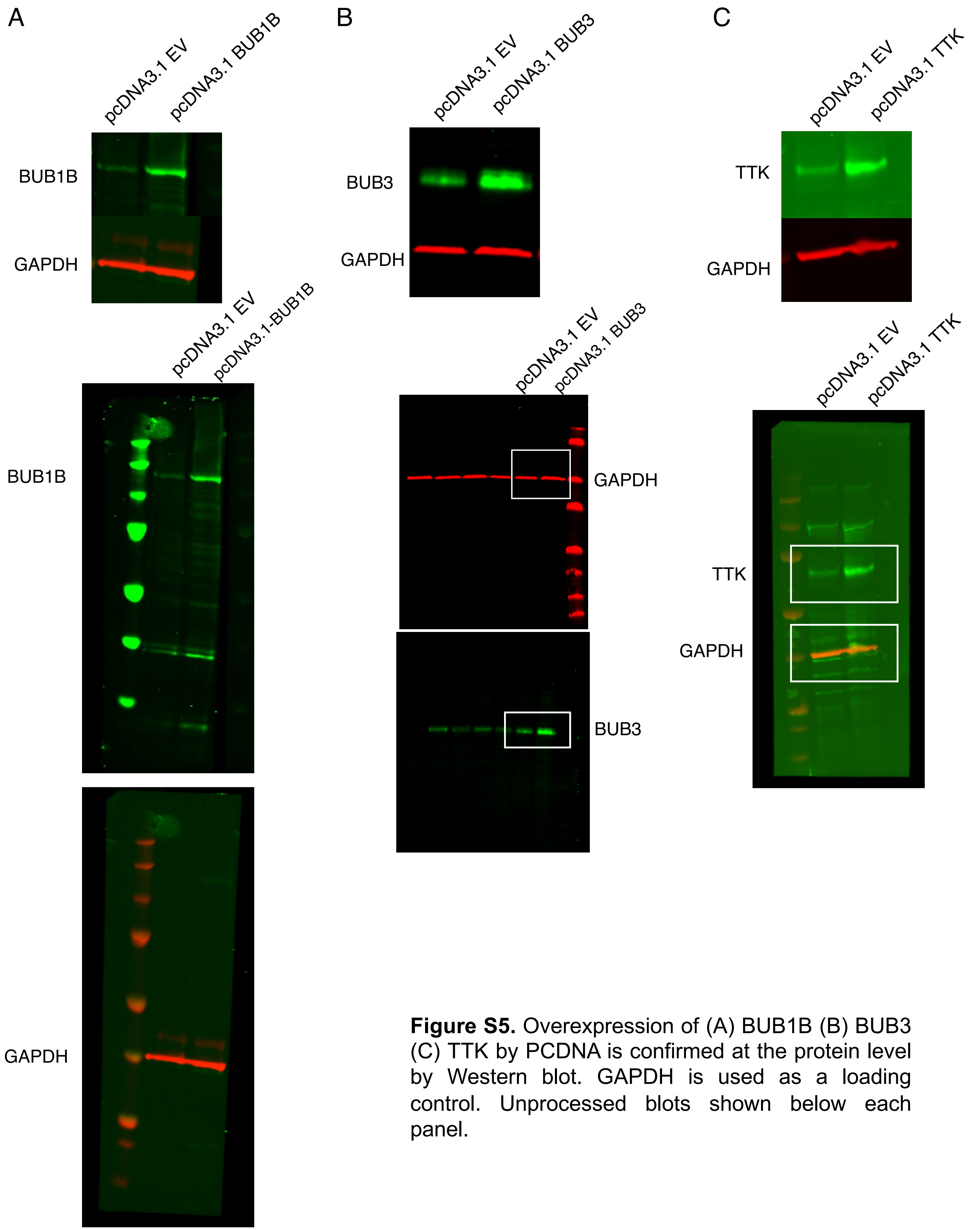

Figure S5. Overexpression of (A) BUB1B (B) BUB3 (C) TTK by PCDNA is confirmed at the protein level by Western blot. GAPDH is used as a loading control. Unprocessed blots shown below each panel.
